# AMPK Activation of Flavonoids from *Psidium guajava* Leaves in L6 Rat Myoblast Cells and L02 Human Hepatic Cells

**DOI:** 10.1155/2019/9209043

**Published:** 2019-12-23

**Authors:** Jiankuan Li, Yujing Zhao, Lingya Cao, Qinghong Zheng, Jianping Gao

**Affiliations:** ^1^School of Pharmaceutical Science, Shanxi Medical University, Taiyuan 030001, China; ^2^School of Basic Medical Science, Shanxi Medical University, Taiyuan 030001, China

## Abstract

**Objective:**

To isolate the hypoglycemic bioactive components from leaves of *Psidium guajava* and evaluate their AMP-activated protein kinase (AMPK) activities.

**Methods:**

A variety of column chromatography was used for the isolation of compounds, and nuclear magnetic resonance (NMR) and mass spectrum (MS) were used for the structure identification of compounds. AMP-activated protein kinase (AMPK) activity of compounds obtained from leaves of *Psidium guajava* was evaluated in L6 rat myoblast cells and L02 human hepatic cells by western blot.

**Results:**

Six principal flavonoids largely present in the leaves of *Psidium guajava*, quercetin (1), quercetin-3-*O*-*α*-L-arabinofuranoside (2), quercetin-3-*O*-*α*-L-arabinopyranoside (3), quercetin-3-*O*-*β*-D-galactopyranoside (4), quercetin-3-*O*-*β*-D-glucopyranoside (5), and quercetin-3-*O*-*β*-D-xylopyranoside (6), were obtained and compound 1–6 exhibited significant activity on AMPK activation both in L6 cells and L02 cells (*p* < 0.01) compared with Control. In particular, the effects of quercetin on AMPK activation were extremely significant compared with Control (*p* < 0.001).

**Conclusions:**

These findings demonstrated that these flavonoids had potential for the activation of AMPK and hypoglycemic activity.

## 1. Introduction

*Psidium guajava* Linn., commonly called guava, is an important plant growing in tropical and subtropical areas in the world. The leaves of *Psidium guajava* are frequently reported to exhibit antidiabetic activity *in vivo* [1–5]. AMPK has been revealed to be an evolutionarily conserved regulator of cellular energy homeostasis in mammalian cells and to be a potential target for energy metabolism-related diseases including type 2 diabetes mellitus [6, 7]. AMPK activation can promote glucose transport and absorption, sugar hydrolysis, and fatty acid oxidation and can inhibit cholesterol and triglyceride synthesis [6–9]. Metformin is a widely used drug for the treatment of type 2 diabetes. Its glucose-lowering effect results from decreased hepatic glucose production and increased skeletal myocyte glucose utilization. The mechanisms of metformin action are poorly understood. Increasing data have implicated that AMPK activation as a mediator is involved in metformin action. Metformin activates AMPK in a number of cell types including myocytes and hepatic cells and tissues, which results in decreased hepatic glucose production and elevated glucose utilization, reduced acetyl-CoA carboxylase (ACC) activity and fatty acid synthesis, and increased insulin sensitivity [10, 11]. Recently, it was reported that the aqueous extract of *Psidium guajava* leaves exhibited the effects of inhibition of hepatic gluconeogenesis and elevation of glycogen synthesis via AMPK/ACC signaling pathways in streptozotocin-induced diabetic rats [12]. In the present research, six main flavonoids were obtained from the aqueous extract of *Psidium guajava* leaves and their activities of AMPK activation were evaluated in both L6 rat myoblast cells and L02 human hepatic cells.

## 2. Methods

### 2.1. General

Silica gel (200–300 mesh, Qingdao Haiyang Chemical Co., Ltd, Qingdao, China), D101 macroporous resin (Tianjin Haiguang Chemical Industry Co., Ltd, Tianjin, China), reversed phase silica gel (ODS 75 *μ*m, YMC Co., Kyoto, Japan), and Sephadex LH-20 (GE Healthcare, Sweden) were used for open column chromatography. Thin layer chromatography (TLC) was performed on silica gel GF_254_(Qingdao Haiyang Chemical Co., Ltd, Qingdao, China). Other chemical reagents were purchased from Damao Chemical Reagent Factory (Tianjin, China). Nuclear magnetic resonance (NMR) analysis was conducted on an AVANCE III 400 MHz (Bruker, Karlsruhe, Germany). Mass spectrum (MS) was performed on an ESI-MS ZQ-2000 (Waters, Massachusetts, America).

L6 rat myoblast cell line (GNR) was purchased from Cell Bank of the Chinese Academy of Science (Shanghai, China) and HL-7702 cell line (CL-0111, L02) was purchased from Procell Life Science and Technology Co., Ltd. (Wuhan, China). Antibodies for phospho-AMPK*α*1 (T183), phospho-AMPK*α*2 (T172) (ab133448), and AMPK*α* (ab131512) were purchased from Abcam (Cambridge, UK). GAPDH (2118) was obtained from Cell Signaling Technology (Beverly, MA, USA). Antibody for horseradish peroxidase (HRP) conjugated secondary goat anti-rabbit antibody (PAB160011) was purchased from CoWin Biotech Co., Ltd. (Beijing, China). Radio-Immuno-Precipitation Assay (RIPA) lysis buffer (P0013C) and bicinchoninic acid assay (BCA) kit (P0010) were purchased from Beyotime Institute of Biotechnology (Shanghai, China).

### 2.2. Plant Material

*Psidium guajava* Linn. leaves were purchased from Bozhou herbal market (Bozhou, China) and was identified by Professor Yune Bai (School of Pharmaceutical Science, Shanxi Medical University). A voucher specimen (SXMU-20160023) was deposited at Herbarium, School of Pharmaceutical Science, Shanxi Medical University.

### 2.3. Extraction and Isolation

The leaves of *Psidium guajava* Linn. (2 kg) were cut into pieces and extracted twice in hot water (80°C) for 1.5 h each time and concentrated to dryness in vacuum at 60°C to obtain the water extract (420 g). The extract was resuspended in water (4 L) and then partitioned successively with petroleum ether, ethyl acetate, and n-butanol. The ethyl acetate fraction (63.1 g) was subjected to silica gel column chromatography eluted with a gradient solvent system of CH_2_Cl_2_-MeOH (10 : 1, 9 : 1, 8 : 2, 7 : 3 v/v) to get four fractions (E-I, II, III, IV). E-III was repeatedly chromatographed with silica gel column chromatography eluted with a gradient solvent system of CH_2_Cl_2_-MeOH (20 : 1, 15 : 1, 10 : 1, 9 : 1 v/v) combined with Sephadex LH-20 column chromatography eluted with 75% methanol to obtain compounds 1 (28.7 mg), 2 (23.5 mg), and 3 (24.6 mg). The n-butanol fraction (83.4 g) was subjected to D101 macroporous resin column chromatography eluted with a gradient solvent system of MeOH-H_2_O (10%, 30%, 50%, 70%, 90%, 100%) to get four fractions (B-I, II, III, IV). B-II was subjected to ODS column chromatography eluted with a gradient solvent system of MeOH-H_2_O (30%, 50%, 70%) combined with repeated silica gel column chromatography eluted with CH_2_Cl_2_-MeOH (10 : 1, 9 : 1 8 : 2 v/v) to obtain compounds 4 (21.8 mg) and 5 (16.6 mg). B-III was subjected to ODS column chromatography eluted with a gradient solvent system of MeOH-H_2_O (30%, 50%, 70%) combined with repeated silica gel column chromatography eluted with CH_2_Cl_2_-MeOH (10 : 1, 9 : 1 8 : 2 v/v) to obtain compound 6 (13.8 mg).

### 2.4. Evaluation of AMPK Activation

L6 cells and L02 cells were treated with compound 1–6 (50 *μ*mol/L) and water extract (100 *μ*mol/L, calculated by quercetin) for 1 h. Metformin (50 *μ*mol/L) and AICAR (4 mmol/L) were used as positive control. The cells untreated with compound 1–6, metformin, or AICAR were considered as Control. Then the cells were washed twice with ice-cold PBS and harvested with 0.25% trypsin. The cells were centrifuged at 1000 rpm for 5 min and washed with ice-cold PBS 3 times and then suspended in 100 *μ*L ice-cold RIPA lysis buffer, sonicated 10 times for 5 s with 10 s pauses in an ice-water bath, and centrifuged at 10,000 rpm for 10 min at 4°C. Protein quantity was detected with BCA assay kit, and an equal amount of proteins was used for western blot analysis.

For western blot analysis, equal amounts of protein extracts (10 *μ*g) were separated by 12% SDS-polyacrylamide gels, and then proteins were transferred onto PVDF membrane (PALL Gelman Laboratory, USA). The membrane was blocked in 5% nonfat milk powder in Tris-buffered saline/0.1% Tween-20 (TBST) for 1.5 h at room temperature and then incubated overnight at 4°C with the primary antibodies. After three washes with TBST, the membrane was incubated for 1 h with horseradish peroxidase (HRP) conjugated goat anti-rabbit IgG as the secondary antibody at room temperature. After three-time washes, the immune blots were detected by enhanced chemiluminescence (ECL) detection kit (CoWin Biotech Co., Ltd., Beijing, China). The NC membrane was analyzed by a TANON GIS-1000 digital gel image analysis system.

### 2.5. Statistical Analysis

Each experiment was performed three times. All data were expressed as mean ± SD. Statistical analysis was performed using the Student's *t*-test and was prepared on Microsoft Office Excel 2003 software. The difference at *p* < 0.05 was indicated to be statistically significant.

## 3. Results

### 3.1. Structure Identification of Compounds Obtained from *Psidium guajava* Leaves

Compounds 1–6 were confirmed as quercetin (1), quercetin-3-*O*-*α*-L-arabinofuranoside (2), quercetin-3-*O*-*α*-L-arabinopyranoside (3), quercetin-3-*O*-*β*-D-galactopyranoside (4), quercetin-3-*O*-*β*-D-glucopyranoside (5), and quercetin-3-*O*-*β*-D-xylopyranoside (6) according to references by ^1^H-NMR, ^13^C-NMR, and MS [13–16].

#### 3.1.1. Compound 1

^1^H NMR (400 MHz, DMSO-*d*_6_): 6.20 (1H, d, *J* = 2.5 Hz, H-6), 6.42 (1H, d, *J* = 2.5 Hz, H-8), 6.90 (1H, d, *J* = 8.0, H-5′), 7.55 (1H, dd, *J* = 8.0 Hz, 2.0 Hz, H-6′), 7.70 (1H, d, *J* = 2.0 Hz, H-2′). ^13^C NMR (100 MHz DMSO-*d*_6_): 93.8 (CH, C-8), 98.7 (CH, C-6), 103.5 (C, C-10), 115.5 (C, C-2′), 116.1 (CH, C-5′), 120.5 (CH, C-6′), 122.4 (C, C-1′), 136.2 (C, C-3), 145.5 (C, C-3′), 147.2 (C, C-2), 148.1 (C, C-4′), 156.6 (C, C-9), 161.2 (C, C-5), 164.3 (C, C-7), 178.2 (C, C-4). ESI-MS: *m/z* (%) = 301.2 [M-H]^−^ (100). Molecular formula: C_15_H_10_O_7_.

#### 3.1.2. Compound 2

^1^H NMR (400 MHz, DMSO-*d*_6_): 5.62 (1H, d, *J* = 4.0 Hz, H-1″), 6.24 (1H, d, *J* = 2.5 Hz, H-6), 6.44 (1H, d, *J* = 2.5 Hz, H-8), 6.90 (1H, d, *J* = 8.0, H-5′), 7.51 (1H, dd, *J* = 8.0 Hz, 2.0 Hz, H-6′), 7.58 (1H, d, *J* = 2.0 Hz, H-2′). ^13^C NMR (100 MHz DMSO-*d*_6_): 61.0 (CH_2_, C-5″), 77.3 (CH, C-3″), 82.5 (CH, C-2″), 86.2 (CH, C-4″), 94.0 (CH, C-8), 99.1 (CH, C-6), 104.4 (C, C-10), 108.2 (CH, C-1″), 116.2 (C, C-2′), 115.9 (CH, C-5′), 122.1 (CH, C-6′), 121.4 (C, C-1′), 133.8 (C, C-3), 145.5 (C, C-3′), 157.4 (C, C-2), 148.9 (C, C-4′), 156.7 (C, C-9), 161.6 (C, C-5), 164.7 (C, C-7), 178.1 (C, C-4). ESI-MS: *m/z* (%) = 433.2 [M-H]^−^ (100). Molecular formula: C_20_H_18_O_11_.

#### 3.1.3. Compound 3

^1^H NMR (400 MHz, DMSO-*d*_6_): 5.62 (1H, d, *J* = 4.0 Hz, H-1″), 6.22 (1H, d, *J* = 2.5 Hz, H-6), 6.42 (1H, d, *J* = 2.5 Hz, H-8), 6.87 (1H, d, *J* = 8.0, H-5′), 7.52 (1H, dd, *J* = 8.0 Hz, 2.0 Hz, H-6′), 7.69 (1H, d, *J* = 2.0 Hz, H-2′). ^13^C NMR (100 MHz DMSO-*d*_6_): 64.8 (CH_2_, C-5″), 66.6 (CH, C-4″), 71.2 (CH, C-2″), 72.2 (CH, C-3″), 93.9 (CH, C-8), 99.1 (CH, C-6), 101.8 (CH, C-1″), 104.3 (C, C-10), 116.0 (C, C-2′), 115.8 (CH, C-5′), 122.5 (CH, C-6′), 121.3 (C, C-1′), 134.1 (C, C-3), 145.4 (C, C-3′), 156.8 (C, C-2), 149.0 (C, C-4′), 156.7 (C, C-9), 161.6 (C, C-5), 164.6 (C, C-7), 177.9 (C, C-4). ESI-MS: *m/z* (%) = 433.2 [M-H]^−^ (100). Molecular formula: C_20_H_18_O_11_.

#### 3.1.4. Compound 4

^1^H NMR (400 MHz, DMSO-*d*_6_): 5.40 (1H, d, *J* = 8.0 Hz, H-1″), 6.21 (1H, d, *J* = 2.5 Hz, H-6), 6.41 (1H, d, *J* = 2.5 Hz, H-8), 6.82 (1H, d, *J* = 8.0, H-5′), 7.52 (1H, dd, *J* = 8.0 Hz, 2.0 Hz, H-6′), 7.69 (1H, d, *J* = 2.0 Hz, H-2′). ^13^C NMR (100 MHz DMSO-*d*_6_): 60.6 (CH_2_, C-6″), 68.4 (CH, C-4″), 71.7 (CH, C-2″), 73.6 (CH, C-3″), 76.3 (CH, C-5″), 94.0 (CH, C-8), 99.1 (CH, C-6), 102.2 (CH, C-1″), 104.4 (C, C-10), 116.4 (C, C-2′), 115.6 (CH, C-5′), 122.1 (CH, C-6′), 121.5 (C, C-1′), 133.9 (C, C-3), 145.3 (C, C-3′), 156.8 (C, C-2), 148.9 (C, C-4′), 156.7 (C, C-9), 161.7 (C, C-5), 164.6 (C, C-7), 177.9 (C, C-4). ESI-MS: *m/z* (%) = 463.1 [M-H]^−^ (100). Molecular formula: C_21_H_20_O_12_.

#### 3.1.5. Compound 5

^1^H NMR (400 MHz, DMSO-*d*_6_): 5.48 (1H, d, *J* = 8.0 Hz, H-1″), 6.21 (1H, d, *J* = 2.5 Hz, H-6), 6.41 (1H, d, *J* = 2.5 Hz, H-8), 6.86 (1H, d, *J* = 8.0, H-5′), 7.58 (1H, dd, *J* = 8.0 Hz, 2.0 Hz, H-6′), 7.69 (1H, d, *J* = 2.0 Hz, H-2′). ^13^C NMR (100 MHz DMSO-*d*_6_): 61.4 (CH_2_, C-6″), 70.4 (CH, C-4″), 74.5 (CH, C-2″), 76.9 (CH, C-5″), 78.1 (CH, C-3″), 94.0 (CH, C-8), 99.1 (CH, C-6), 101.3 (CH, C-1″), 104.5 (C, C-10), 116.6 (C, C-2′), 115.4 (CH, C-5′), 122.1 (CH, C-6′), 121.6 (C, C-1′), 133.7 (C, C-3), 145.3 (C, C-3′), 156.6 (C, C-2), 148.9 (C, C-4′), 156.7 (C, C-9), 161.7 (C, C-5), 164.6 (C, C-7), 177.9 (C, C-4). ESI-MS: *m/z* (%) = 463.2 [M-H]^−^ (100). Molecular formula: C_21_H_20_O_12_.

#### 3.1.6. Compound 6

^1^H NMR (400 MHz, DMSO-*d*_6_): 5.14 (1H, d, *J* = 8.0 Hz, H-1″), 6.21 (1H, d, *J* = 2.5 Hz, H-6), 6.42 (1H, d, *J* = 2.5 Hz, H-8), 6.85 (1H, d, *J* = 8.0, H-5′), 7.58 (1H, dd, *J* = 8.0 Hz, 2.0 Hz, H-6′), 7.68 (1H, d, *J* = 2.0 Hz, H-2′). ^13^C NMR (100 MHz DMSO-*d*_6_): 66.5 (CH2, C-5″), 69.8 (CH, C-4″), 74.0 (CH, C-2″), 76.5 (CH, C-3″), 94.0 (CH, C-8), 99.1 (CH, C-6), 102.2 (CH, C-1″), 104.4 (C, C-10), 116.6 (C, C-2′), 115.8 (CH, C-5′), 121.9 (CH, C-6′), 121.3 (C, C-1′), 133.6 (C, C-3), 145.4 (C, C-3′), 156.7 (C, C-2), 148.9 (C, C-4′), 156.7 (C, C-9), 161.7 (C, C-5), 164.6 (C, C-7), 177.8 (C, C-4). ESI-MS: *m/z* (%) = 433.1 [M-H]^−^ (100). Molecular formula: C_20_H_18_O_11_.

### 3.2. AMPK Activities of Compounds in L6 Rat Myoblast Cells

**Figure 1:**
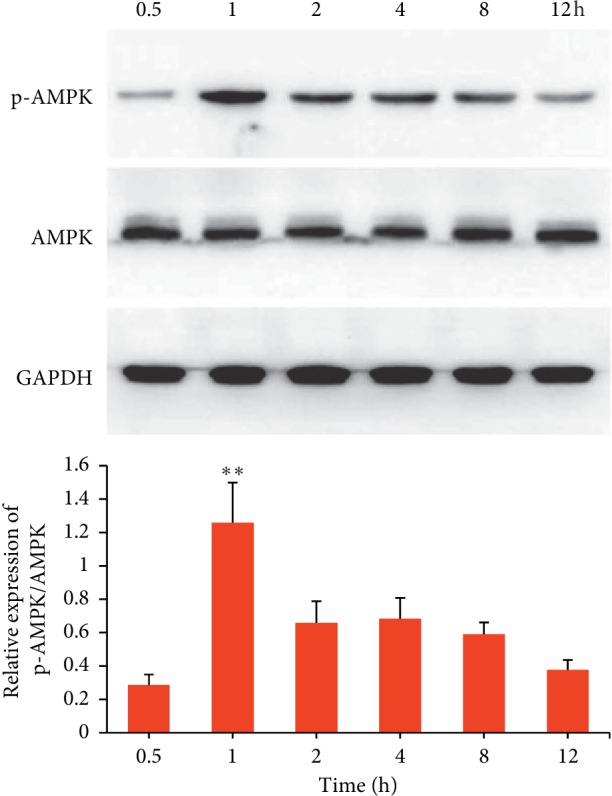
AMPK activation in L6 cells treated by quercetin (50 *μ*mol/L) for 0.5, 1, 2, 4, 8, and 12 h. Data are presented as mean ± SD (*n* = 3). ^*∗∗*^*p* < 0.001 versus 0.5, 2, 4, 8, and 12 h.

As shown in Figure 1, AMPK activation (phosphorylation) reached its maximum level when the L6 rat myoblast cells were treated by quercetin (50 *μ*mol/L) for 1 h, and then the activation of AMPK faded slowly over time. So, in the subsequent evaluation of AMPK activation, the treatment time was confirmed to be 1 h.

**Figure 2:**
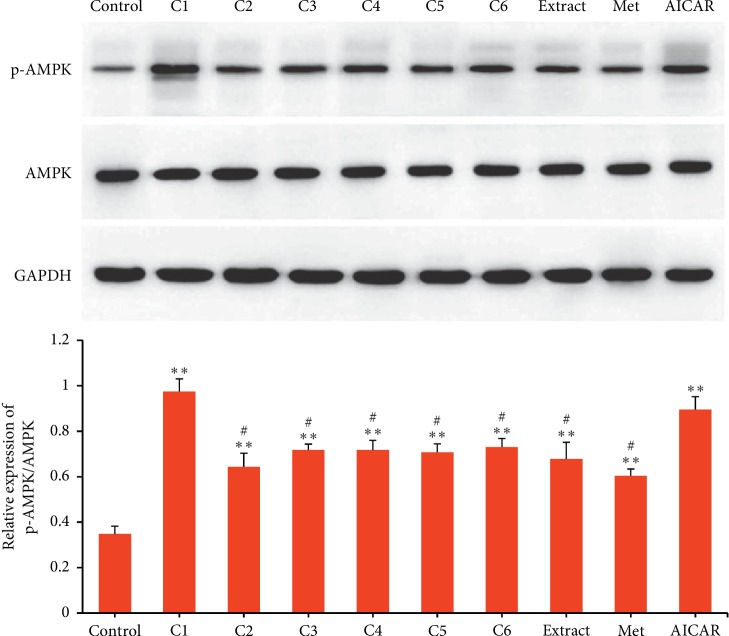
AMPK activation in L6 cells activated by compound 1–6 (C1–C6), water extract (Extract), metformin (Met), and AICAR. Data are presented as mean ± SD (*n* = 3). ^*∗∗*^*p* < 0.001 versus Control, ^#^*p* < 0.05 versus C1 and AICAR.

As shown in Figure 2, the effects of compounds 1–6, water extract, metformin, and AICAR on AMPK activation were evaluated in L6 rat myoblast cells. It was shown that compounds 1–6, water extract, metformin, and AICAR exhibited significant AMPK activation in L6 rat myoblast cells compared with Control (*p* < 0.001). Furthermore, it was observed that compound 1 and AICAR showed greater effects on AMPK activation than that of compounds 2–6 (*p* < 0.05). There was no significant difference in AMPK activation among compounds 2–6 and metformin.

### 3.3. AMPK Activities of Compounds in L02 Human Hepatic Cells

**Figure 3:**
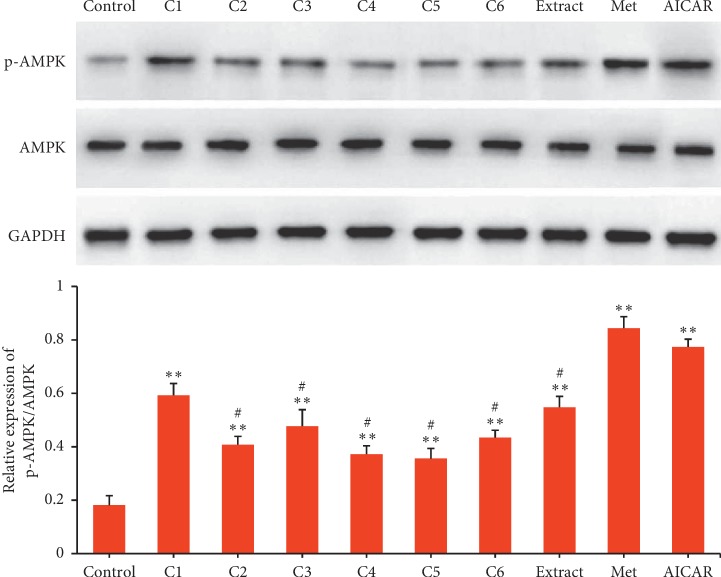
AMPK activation in L02 cells activated by compound 1–6 (C1–C6), water extract (Extract), metformin (Met), and AICAR. Data are presented as mean ± SD (*n* = 3). ^*∗∗*^*p* < 0.001 versus Control, ^#^*p* < 0.05 versus C1, Met and AICAR.

As shown in Figure 3, the effects of compounds 1–6, water extract, metformin, and AICAR on AMPK activation were evaluated in L02 human hepatic cells. The result showed that compounds 1–6, water extract, metformin, and AICAR exhibited significant AMPK activations in L02 human hepatic cells compared with Control (*p* < 0.001). Also, it was observed that compound 1, metformin, and AICAR exhibited greater effects than that of compounds 2–6 in L02 human hepatic cells (*p* < 0.05). Meanwhile, quercetin exhibited higher activity than quercetin glycosides (compound 2–6), which was consistent with the result obtained in L6 rat myoblast cells. So, it could be concluded that the OH substitution at C-3 position of quercetin was very necessary for AMPK activation.

## 4. Discussion

Flavonoids are frequently present in vegetables and fruits and the consumption of flavonoids-rich diet is accepted to be beneficial against metabolic diseases including obesity, diabetic mellitus, and insulin resistance [17, 18]. *Psidium guajava* leaves are often reported to exhibit antidiabetic effect as well as antioxidant, anticoagulant, anti-inflammatory, and immune-stimulatory effects [19–22]. In the present research, we obtained six flavonoids from *Psidium guajava* leaves, which were also reported in previous studies [13–16]. These data indicated that flavonoids especially quercetin and quercetin glycosides were the main components presented in *Psidium guajava* leaves. AMPK activation is considered to be a key event in glucose metabolism, and AMPK phosphorylation level in threonine 172 is accepted to be a marker of AMPK activation [6, 7, 23]. AMPK has been accepted to be a potential target for energy metabolism-related diseases including type 2 diabetes mellitus [6–9]. Eid et al. reported that quercetin and quercetin glycosides were the main active principles of *Vaccinium vitis-idaea* for antidiabetic effect by activating AMPK in muscle cells [24]. They found that quercetin, quercetin-3-glucoside, and quercetin-3-galactoside at 50 *μ*mol/L and 100 *μ*mol/L exhibited the activation on AMPK effector ACC in C2C12 murine skeletal cells. Our present research demonstrated that quercetin and quercetin glycosides (1–6) at 50 *μ*mol/L induced the phosphorylation of AMPK in both L6 rat myoblast cells and L02 human hepatic cells. Both studies obtained similar results that quercetin and quercetin glycosides had the potential of activating AMPK signaling pathway in C2C12, L6, and L02 cells. However, our present study demonstrated that the aglycone quercetin exhibited stronger effects than those of quercetin glycosides on the AMPK activation in both L6 rat myoblast cells and L02 human hepatic cells (*p* < 0.01), which indicated that the OH substitution at C-3 of quercetin was required for AMPK activation. Recently, it was also reported that quercetin-rich guava juice in combination with Trehalose exhibited antidiabetic effect in type 2 diabetic rats [25]. Therefore, it was suggested that quercetin and quercetin glycosides from *Psidium guajava* leaves were likely responsible for the antidiabetic effect of the leaves of *Psidium guajava*.

## 5. Conclusion

The findings demonstrated that quercetin and its glycosides from *Psidium guajava* leaves exhibited significant AMPK activity and were likely responsible for the antidiabetic effect of *Psidium guajava* leaves.

## Data Availability

The data used to support the findings of this study are available from the corresponding author upon request.
